# Diagnosis of orthostatic tremor using smartphone accelerometry

**DOI:** 10.1186/s12883-021-02486-0

**Published:** 2021-11-22

**Authors:** Nicholas E. Calvo, Joseph M. Ferrara

**Affiliations:** 1 Division of Neurology, Virginia Tech Carilion School of Medicine, 3 Riverside Circle, Roanoke, Virginia, 24016 USA; 2 Department of Neurology, University of South Carolina School of Medicine, 8 Medical Park, Suite 420, Columbia, SC 29203 USA

**Keywords:** Accelerometry, Movement disorders, Neurology, Orthostatic tremor, Smartphone

## Abstract

**Background:**

Primary orthostatic tremor (OT) is a rare movement disorder characterized by a 13–18 Hz leg tremor, which arises when standing and is relieved by walking/sitting. Those affected generally do not fall, but experience fear of falling, lessened by ambulation. Because of its low amplitude, the tremor is not readily visible, and diagnosis requires confirmation with surface electromyography (sEMG). Recently, applications using the accelerometer feature of smartphones have been used to detect and quantify tremors, including OT, though the accuracy of smartphone accelerometry (SPA) in diagnosing OT is unknown.

**Methods:**

We completed SPA in consecutive adults (18+ years), who presented to our neurology clinic with either subjective leg shakiness upon standing or unsteadiness when standing that lessened with ambulation, which comprised 59 of 2578 patients. We assessed tremor using the StudyMyTremor application on an iPhone 6 s adhered with tape to the patient’s tibialis anterior. Surface electromyography was completed on the same muscle. The primary outcome of this study was to determine SPA’s sensitivity and specificity in detecting OT compared with surface electromyography.

**Results:**

Fifty-nine patients with the following diagnoses were included: OT (6), Parkinson’s disease, Hereditary Spastic Paraplegia, orthostatic hypotension, essential tremor, spinal cerebellar ataxia, sensory ataxia and functional movement disorder. Smartphone accelerometry detected a 13–18 Hz tremor in 5 of 6 patients diagnosed with OT by sEMG with no false positives in other conditions, yielding a sensitivity of 83%, specificity of 100% in the cohort we studied.

**Conclusions:**

Though a larger sample size is desirable, preliminary data suggest that smartphone accelerometry is an alternative to surface electromyography in diagnosing OT.

**Supplementary Information:**

The online version contains supplementary material available at 10.1186/s12883-021-02486-0.

## Background

Orthostatic tremor (OT) is a rare movement disorder characterized by a low amplitude, high frequency (13–18 Hz) leg tremor which arises when standing, improves with walking, and remits rapidly when lying or sitting. Those affected experience unsteadiness when standing and associated fear of falling, but rarely fall. Nonetheless, because stationary stance cannot be maintained, patients with OT experience impairments in health-related quality measures commensurate to Parkinson Disease [1]. Treatment with clonazepam or gabapentin provides some benefit in the majority of patients [2].

A diagnosis of OT should be suspected in any patient whose unsteadiness improves with walking; however, the tremor often cannot be visualized because of its low amplitude, and confirmation of the diagnosis requires electrophysiological testing to determine tremor frequency, such as surface electromyography (sEMG). Historically, diagnostic delays are common, averaging approximately 7–9 years [1, 2]. In the primary care setting, lack of familiarity and lack of timely access to electrophysiological testing may contribute to diagnostic delays.

Recently, applications using the accelerometer within smartphones (SPA) have been used to detect and quantify tremors, including OT [3, 4], though the accuracy of SPA in distinguishing OT from alternate causes of instability is unknown. In our study, we assessed the sensitivity and specificity of SPAs in detecting OT, when compared with sEMG, with the goal of facilitating diagnostic options for the primary care provider.

## Methods

We completed SPA in consecutive adults (18+ years) who presented/returned to our movement disorder neurology clinic with either “leg shakiness while standing” or “unsteadiness while standing that improves with walking”. Patients who were unable to stand for 1 min were excluded from the study. We recruited patients over an 18-month interval. This study was approved by Carilion Clinic Institutional Review Board (IRB#2505), participants provided informed consent and methods were performed in accordance with institutional research regulations.

**Fig. 1:**
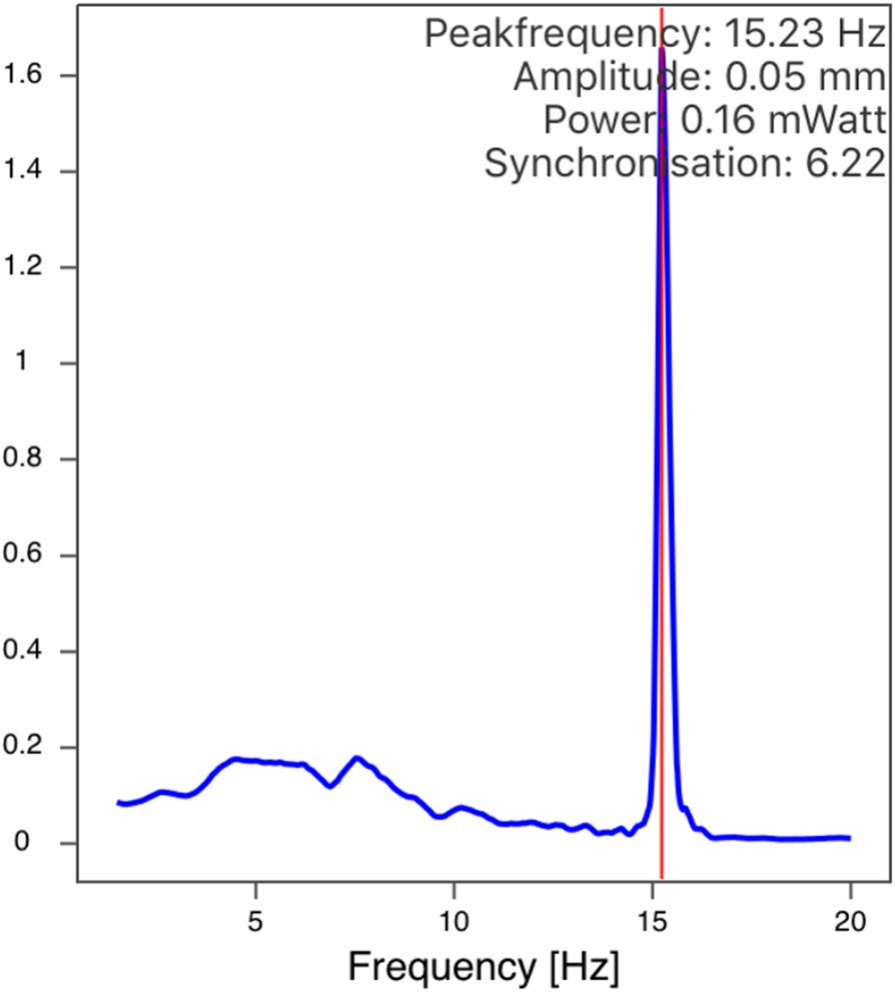
The StudyMyTremor application display showing the numerical peak frequency based on spectrum analysis of 15.32 Hz, characteristic of OT

We assessed for tremor using the StudyMyTremor application on an iPhone 6 s. The application provides a numerical peak frequency based on spectral analysis (Fig. 1).

**Fig. 2:**
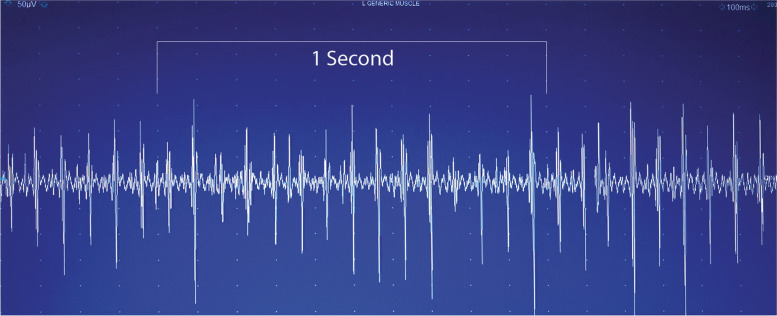
SEMG recording showing a 16 Hz tremor over a 1 s interval consistent with OT

We adhered the phone over the patient’s right tibialis anterior with medical tape and a one-minute recording was completed using default settings of the application. SPA was recorded while patients stood at a self-selected comfortable stance width. sEMG was performed on the same muscle immediately prior to SPA. sEMG was analyzed visually and auditorily for tremor and if present tremor frequency was calculated over a 1 s interval (Fig. 2, Suppl Video).

**Additional file 2**: **Supplemental Video**: A video showing SEMG free run with the gain set to 50 uv/div and a sweep speed of 100 msec/div.

Orthostatic vital signs and a comprehensive neurological examination were also completed.

The primary outcome of this study was to determine the sensitivity and specificity of SPA in detecting OT (defined as a 13–18 Hz peak frequency) when compared with SEMG as the gold standard test.

## Results

Fifty nine of 2578 consecutive patients met inclusion criteria. The study population’s mean age was 65 +/− 14 years and 34 were women. Clinical diagnoses are displayed in Table 1. SPA detected a 13–18 Hz tremor in 5 of 6 patients diagnosed with OT by sEMG with no false positives in other conditions, yielding a sensitivity of 83%, specificity of 100% in the cohort we studied.

**Table 1:** Diagnoses of Study Participants

Diagnosis	Number of Patients
Parkinson’s Disease	9
Essential tremor	7
Whole body tremulousness from medication-related tremor or polymyoclonus	7
OT	6
Higher level gait disorder related to small vessel ischemic disease or ventriculomegaly	5
Orthostatic hypotension	5
Cerebellar ataxia	5
Sensory ataxia from neuropathy	3
Spasticity with clonus	3
Drug-induced parkinsonism	2
Functional movement disorder	2
Vibratory paresthesias or tremulous sensation of unknown cause	2
Stiff person syndrome	1
Postural orthostatic tachycardia syndrome	1
Possible vestibulopathy	1

Legend: Clinical diagnoses of patients meeting inclusion criteria. The study included adults presenting to a subspecialty movement disorder clinic who reported either “leg shakiness while standing” or “unsteadiness while standing that improves with walking

## Discussion

This study suggests that SPA can rapidly and accurately distinguish OT from other disorders of static balance. Given the ubiquitous nature of smartphones, ease of diagnostic assembly, and low cost, we anticipate that SPA will be useful in diagnosing OT in the primary care setting where sEMG is not readily available. We hope that access to diagnostics will also improve awareness of this disabling and under-recognized disorder. Limitations of our study include the relatively small number of patients with OT, a consequence of its rarity. We assessed for tremor over the tibialis anterior, while others adhered SPA over the ankle or thigh. The best recording site for SPA in OT is not known, but OT can be measured with sEMG over a variety of limb, axial and even cranial muscles [5], making it unlikely that exact positioning for SPA is of critical importance. We used a smartphone model that was readily available at the initiation of this study. Nonetheless, smartphones and their software are in a state of constant evolution and changes in technology may impact future implementation. Multiple SPA applications are now available to measure tremor and only limited data is published as to how they might differ. We used the StudyMyTremor application, which can be obtained at low cost, has been formally studied in another tremor population, and displays a visible frequency power spectrum and numeric peak frequency [6]. Because this application records tremor over a 1 min interval, we excluded 2 patients who were unable to stand for that 1 min. Neither individual had evidence of OT on sEMG, but some patients with severe OT may be unable to stand for a duration suitable for SPA using the StudyMyTremor application. To facilitate ease of use in the clinic setting, we defined a positive result as a peak frequency between 13 and 18 hz, though we advise reviewing the spectra analysis as the single OT patient in our study who went undetected with SPA did have a smaller peak in the OT range (Suppl Fig. 1). Peaks at multiple frequencies can represent subharmonic oscillations or the presence of multiple tremor types. In such cases confirmation with sEMG remains a necessity. We calculated sEMG tremor frequency over a 1 s interval but did not complete fast Fourier transformation on sEMG data, so differences in peak frequency between sEMG and SPA cannot be quantified. Finally, we screened for OT by asking patients whether they had “leg shakiness while standing” or “unsteadiness while standing that improves with walking”, but scales for screening or evaluating OT have not yet been validated, and sEMG was not completed on those who did not meet inclusion criteria.

## Supplementary Information

**Additional file 1: Supplemental Figure 1.** The StudyMyTremor application showing multiple numerical peak frequencies based on spectrum analysis. This case initially went undetected with SPA, but spectrum analysis shows there is a smaller peak at 18 Hz (in the OT range).

## Data Availability

All data generated or analyzed during this study are the possession of Virginia Tech Carilion School of Medicine Department of Research and Development and are available from the corresponding author on reasonable request.
