# Supplementary figures and images for: Diagnosis of orthostatic tremor using smartphone accelerometry
Source: BMC Neurol. 2021 Nov 22;21:457. doi: 10.1186/s12883-021-02486-0 (PMC8607557; doi:10.1186/s12883-021-02486-0)

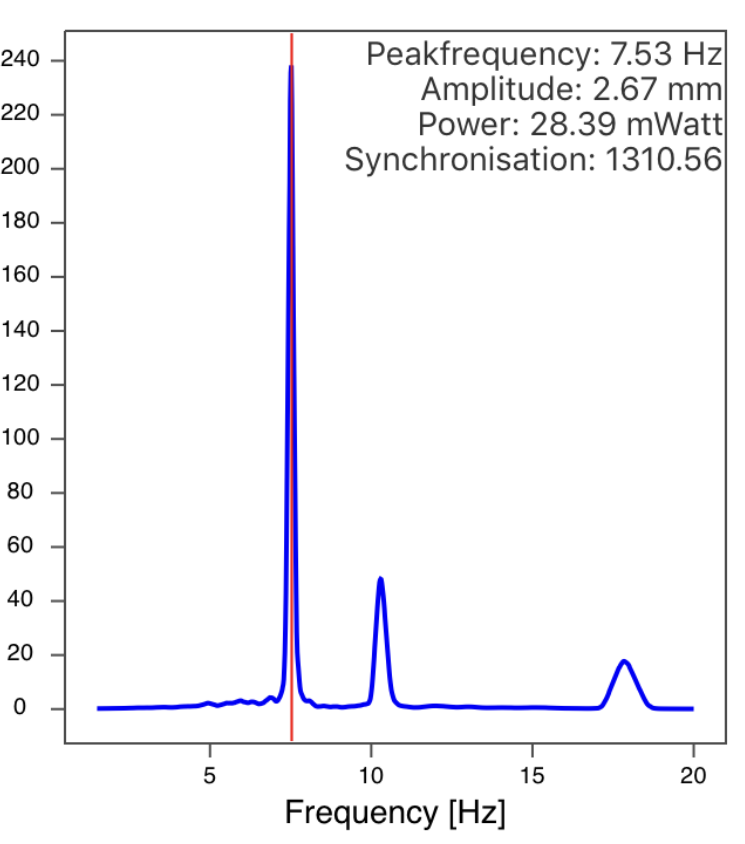

Supplement: Supplementary file 1 — Additional file 1: Supplemental Figure 1. The StudyMyTremor application showing multiple numerical peak frequencies based on spectrum analysis. This case initially went undetected with SPA, but spectrum analysis shows there is a smaller peak at 18 Hz (in the OT range). [file 12883_2021_2486_MOESM1_ESM.zip › Supplemental figure 1.tif]
